# A Comparative Study of Growth Kinetics, *In Vitro* Differentiation Potential and Molecular Characterization of Fetal Adnexa Derived Caprine Mesenchymal Stem Cells

**DOI:** 10.1371/journal.pone.0156821

**Published:** 2016-06-03

**Authors:** Anjali Somal, Irfan A. Bhat, Indu B., Sriti Pandey, Bibhudatta S. K. Panda, Nipuna Thakur, Mihir Sarkar, Vikash Chandra, G. Saikumar, G. Taru Sharma

**Affiliations:** 1 Division of Physiology and Climatology, ICAR-Indian Veterinary Research Institute, Izatnagar-243 122, Bareilly, U.P., India; 2 Division of Veterinary Pathology, ICAR-Indian Veterinary Research Institute, Izatnagar-243 122, Bareilly, U.P., India; Indian Institute of Toxicology Research, INDIA

## Abstract

The present study was conducted with an objective of isolation, *in vitro* expansion, growth kinetics, molecular characterization and *in vitro* differentiation of fetal adnexa derived caprine mesenchymal stem cells. Mid-gestation gravid caprine uteri (2–3 months) were collected from abattoir to derive mesenchymal stem cells (MSCs) from fetal adnexa {amniotic fluid (cAF), amniotic sac (cAS), Wharton’s jelly (cWJ) and cord blood (cCB)} and expanded *in vitro*. These cultured MSCs were used at the 3^rd^ passage (P3) to study growth kinetics, localization as well as molecular expression of specific surface antigens, pluripotency markers and mesenchymal tri-lineage differentiation. In comparison to cAF and cAS MSCs, cWJ and cCB MSCs showed significantly (P<0.05) higher clonogenic potency, faster growth rate and low population doubling (PDT) time. All the four types of MSCs were positive for alkaline phosphatase (AP) and differentiated into chondrogenic, osteogenic, and adipogenic lineages. These stem cells expressed MSC surface antigens (CD73, CD90 and CD105) and pluripotency markers (Oct4, Sox2, Nanog, KLF, cMyc, FoxD3) but did not express CD34, a hematopoietic stem cell marker (HSC) as confirmed by RT-PCR, immunocytochemistry and flow cytometric analysis. The relative mRNA expression of MSC surface antigens (CD73, CD90 and CD105) was significantly (P<0.05) higher in cWJ MSCs compared to the other cell lines. The mRNA expression of Oct4 was significantly (P<0.05) higher in cWJ, whereas mRNA expression of KLF and cMyc was significantly (P<0.05) higher in cWJ and cAF than that of cAS and cCB. The comparative assessment revealed that cWJ MSCs outperformed MSCs from other sources of fetal adnexa in terms of growth kinetics, relative mRNA expression of surface antigens, pluripotency markers and tri-lineage differentiation potential, hence, these MSCs could be used as a preferred source for regenerative medicine.

## Introduction

Over the past decade, stem cell-based regenerative therapy is gaining popularity due to their capacity to renew themselves through mitotic cell division and differentiation into specialized cell types of diverse range [1,2]. Stem cells could be isolated from the embryos, fetuses including adnexa and adult tissues [3]. Embryonic stem cells (ESCs), derived from the inner cell mass of blastocyst are pluripotent cells possessing the ability to differentiate into ectoderm, endoderm and mesoderm [4,5], however, the clinical use of these ESCs is limited, due to their teratogenic nature, allogeneic rejection and ethical issues. MSCs are multipotent stem cells which can be isolated from almost all tissues and can be effectively expanded *in vitro*[6]. Bone marrow (BM) has been the major source of MSCs for treating various ailments in veterinary sciences [7–10]. Bone Marrow-derived MSCs (BM-MSCs) are directly isolated from the animals but the procedure being invasive is painful to the animal [11] and also carries the risk of infection. The estimated mononuclear cells that are MSC from a typical BM aspirate are in the proportion of 0.001–0.01% only [12], moreover, these adult stem cells show reduced plasticity and growth with increasing donor age and multiple passages *in vitro* [13,14]. Thus, fetal adnexa derived stem cells are viewed as a good source of MSCs, which could address the above-mentioned concerns since these stem cells are ethically acceptable, easily accessible and non-invasively procured from deserted and abandoned tissue [15–17]. Further, the yield of stem cells from the porcine umbilical cord (7%) is relatively higher as compared to BM-MSCs (1%) [18]. MSCs derived from fetal adnexa conserve many characteristics of primitive embryonic layers from which they originate and hence express embryonic markers. The *in vitro* culture of these stem cells exhibits extensive proliferative capacity,immense differentiation potential, longest telomere length which lies intermediate between ESCs and multipotent adult stem cells [19–21]. The *in vitro* culture of these gestational tissues maintains the normal karyotypes even at later passages[22]. Fetal adnexa MSCs are non-immunogenic in nature and upon activation secrete high concentration of mediators [23].

Studies are required in a diverse range of animal models to be a source of stem cells and host for allogeneic and xenogeneic tissue grafts to test the safety of promising therapies and establish proof-of-concept. Instead of using laboratory animal models, such as mice, rat, rabbit, large animal species viz. canine, ovine, caprine, porcine and non-human primates could make better models for biomedical research, due to their longer lifespan and relatively more similarities at physiological level with humans. These species provide an advantage for testing stem cell therapies, besides providing different types of stem cells that can be extracted in abundance from a single animal source and manipulated for *in vitro* analysis and various therapeutic applications [24]. Amongst them *Capra hircus*, the domesticated goatis the preferred model in various orthopedic, chemotherapeutic, psychological and physiologic researches [25, 26]. Isolation, culture and characterization of cWJ and cAF MSCs have been reported previously by different workers [27–30], cWJ MSCs have also been investigated in the xenogeneic animal model for their tissue healing potential [30]. Comparative studies on isolation, *in vitro* expansion, growth kinetics, molecular characterization and *in vitro* differentiation potential of different sources of fetal adnexa derived stem cells are available for canine, feline, equine and bovine species [31–34] but not in caprine species. Thus, the present study was conducted with an objective to compare growth kinetics, *in vitro* differentiation potential and molecular characterization of caprine MSCs derived from different types of fetal adnexa.

## Materials and Methods

### Cell isolation, culture and expansion

Gravid uteri of pregnant goats at mid-gestation (2–3 months) were collected from the local abattoir located at Theria Mohanpur, Bareilly, Uttar Pradesh and transported to the laboratory within 2 hrs of slaughter. Approval for this study was granted by the institute animal ethics committee (IAEC) of Indian Council of Agriculture Research (ICAR)-Indian Veterinary Research Institute (IVRI), Izatnagar vide order no. F. 1-53/2012-13-JD (Res) dated 10.09.2013. The fetus was washed for a couple of times with NSS fortified with antibiotics, to remove dirt, blood and other debris, followed by dissection to procure the fetal adnexa.cAF (40–50 ml) was collected from the amniotic cavity in Falcon® tubes and centrifuged at 1000 rpm for 10 min to separate cells from the liquid fraction. These recovered cells were seeded in cell culture plates containing high-glucose DMEM (D5796; Sigma) supplemented with 15% FBS (16000–044, Gibco (Life Technologies, USA)) and 50 μg/ml gentamycin sulphate (G1272; Sigma), and placed in the CO_2_ incubator maintained at 37°C with 5% CO_2_ and maximum humidity. cWJ and cAS were separated from the fetus and cultured *in vitro* by explant method, whereas, cCB was isolated from the umbilical cord, mononuclear cells were separated by density gradient centrifugation and were seeded in growth medium as described above. Media was changed every 4^th^ day, cells were harvested using Accutase® (A6964; Sigma), upon reaching 70–80% confluence and reseeded @ of 2 x 10^4^ cells/cm^2^. A homogenous population of these cultured cells at P3 were used for all the experiments conducted for this study.

### Growth kinetics

To determine the PDT of all the four types of adnexa derived MSCs, cells at P3 were seeded into a 24-well culture plate at a density of 1×10^4^ cells/well and incubated in the growth medium which was changed every 4^th^ day [29]. The cells were harvested daily using Accutase® and the cell number was enumerated with Countess® automated cell counter (Invitrogen Corp., CA, USA) sequentially from 2 wells over 12 consecutive days. The growth curve was generated by calculating the mean number of cells and thereafter plotting a semi-log curve against culture time. PDT was calculated using the equation described by Pratheesh *et al*., [29] i.e. PDT = t log2/log (N_t_/N_0_), where t depicts culture period in hrs, N_0_ depicts initial cell number and N_t_ depicts cell number at particular culture period.

### Colony forming unit assay

The colony forming unit (CFU) assay of MSCs was performed to test the clonogenic potential of isolated cells at the 1^st^ passage (P1). In brief, for this assay, 100 nucleated cells, isolated from caprine MSCs were seeded in three 94 X 16 mm culture dishes (CELLSTAR, Greiner Bio-one, USA) and incubated for a period of two weeks at 37°C in a humidified atmosphere having 5% CO_2_. The medium was completely renewed every fourth day, at the end of incubation, cells were washed twice with DPBS (10010–023; Life Technologies) and fixed in 4% formaldehyde solution followed by 15 minutes staining with 1% crystal violet solution. Each dish was counted under an inverted microscope (Olympus-1X71, Tokyo, Japan), cell clusters containing ≥20 nucleated cells were observed and scored as per the method of Gade *et al*., [35].

### Alkaline phosphatase staining

All the four types of fetal adnexa MSCs at P3 were cultured, till complete confluence and upon establishment of the monolayer, were subjected to AP staining. Growth medium was removed from these cultures and monolayers were washed well with pre-warmed DMEM. AP activity was observed using AP Live Stain (A14353; Molecular Probe, Life Technologies, USA). AP Live stain solution (1X) was applied directly onto the adherent cells and incubated for 30 min at 37°C. After removing the stain, cells were washed twice with DMEM and visualized under a fluorescent microscope (Olympus-IX71, Tokyo, Japan) using standard FITC filter.

### Chondrogenic differentiation

Chondrogenic differentiation was assessed in different cell lines of caprine fetal adnexa using chondrogenic differentiation kit (A10064-01; StemPro, Gibco Life Technologies, USA). The expansion medium was replaced by the chondrogenic differentiation medium after cells attained 60–70% confluence. As a negative control, all the cell lines were also maintained in the expansion medium and cultured for 14 days, media was changed twice a week and then differentiation was demonstrated using Alcian blue 8GX (Sigma-Aldrich) stain [29].

### Osteogenic differentiation

Osteogenic differentiation kit (A10066-01; StemPro, Gibco Life Technologies, USA) was used to assess osteogenic differentiation as per manufacturer’s instructions. The cells were seeded at a density of 1×10^4^ cells/well, once they were at 60–70% confluence; the expansion medium was replaced by the osteogenic differentiation medium. All the cell lines were also maintained in the expansion medium, as a negative control. Cells were cultured for two weeks and the medium was changed twice a week and the differentiation was assessed by Von Kossa staining (1% silver nitrate and 5% sodium thiosulfate) to detect calcium deposits [31].

### Adipogenic differentiation

Adipogenic differentiation kit (A10065-01, StemPro, Gibco Life Technologies, USA) was used as per manufacturer’s instructions for the adipogenic induction of caprine fetal adnexa MSCs. The expansion medium was replaced by the adipogenic differentiation medium after attaining 60–70% confluence. Stem cells from all the four types of sources were cultured for two weeks, media was changed every 4^th^ day and the differentiation was evaluated by the presence of lipid droplets that were observed after staining with oil o red stain [29]. As a negative control, an equal number of cells were maintained in the expansion medium.

### Phenotypic characterization

Phenotypic characterization of *in vitro* cultured fetal adnexa MSCs was done by localizing surface and pluripotency markers by immunocytochemistry and then assessing through flow cytometry.

#### Immunocytochemistry

All the cell lines at P3 were expanded until a minimum confluency of 60–80% and fixed with 4% formaldehyde solution for 20 min at 37°C and then permeabilized with 0.25% Triton-X for 15 min at 37°C. Nonspecific binding was blocked with 5% bovine serum albumin (8806; Sigma) for 45 minutes, cells were probed with different primary antibodies against cell surface markers [CD73 (SC-14682), CD90 (SC-31244), CD105 (SC-57099)] and pluripotency-related transcriptional factors [Oct4 (SC-8628), Nanog (SC-30328), Sox2 (SC-54517), KLF (SC-48570), cMyc (SC-42) and FoxD3 (SC-27888)] at 1:100 dilution for each of them with and overnight incubation period at 4°C. Antigens were localized by incubating these stem cells with FITC (SC-2099) or TR (SC-2783) conjugated donkey anti-goat or donkey anti-mouse secondary antibodies {as the case may be} with a dilution rate of 1: 200 for 2 hrs at 37°C in a dark environment, thereafter counterstained with 4', 6-diamidino-2-phenylindole (DAPI) (SC-3598). Negative controls were processed similarly, except for the exclusion of primary antibodies. Images were captured using the fluorescent microscope (IX 71, Olympus, Shinjuku, Tokyo, Japan).

#### Flow cytometry

The expression of cell surface markers [CD 73^+^, CD 105^+^, and CD34^-^ (SC-7045)] and pluripotency markers (Oct4, Sox2 and Nanog) in caprine fetal adnexa MSCs were analyzed at P3 using flow cytometry as described by Gade *et al*., [35]. Using accutase®, fetal adnexa MSCs were harvested from the confluent monolayer and resuspended in PBS post counting. Cells were pelleted and fixed with 4% formaldehyde solution followed by permeabilization with 0.10% Triton-X. Non-specific binding was blocked by incubating adnexa derived MSCs with blocking sera (from the species in which secondary antibodies were raised) for 30 min. Cells were incubated with primary antibodies at 1:100 dilution for 1 hr followed by another 1 hrincubation with FITC-conjugated secondary antibodies at 1:200 dilution. The cells were finally washed and re-suspended in PBS. The complete procedure was simulated for control samples, except the incubation with primary antibodies. Flow cytometry was performed using the FACS Calibur (BD Biosciences, San Diego, CA, USA) and data analysis with Cell Quest software (BD Biosciences).

#### Total RNA extraction and cDNA synthesis

The total RNA was harvested from all the four cell lines, using TRIzol reagent (90305; Ambion, Life Technologies, USA) at P3. RNA concentration and purity was checked in Nanodrop Spectrophotometer (Thermo Scientific, USA) and the integrity was accessed by 1.5% agarose gel electrophoresis. Samples with A_260/280_ values in between 1.8–2.0 were used for cDNA synthesis. 1 μg of total RNA was used as template for the reverse transcription using Verso cDNA synthesis kit (AB- 1453/B; Thermo Scientific, USA) following the manufacturer’s protocol. The total 20μl reaction volume was subsequently reverse transcribed in Bioer XP cycler PCR machine (Biotron healthcare P. Ltd.) by incubating at 42°C for 59 min followed by the final termination of the reaction by heating for 2 min at 95°C.

#### PCR amplification Quantitative real-time PCR (qPCR) analysis

PCR amplification was done in Bioer XP cycler PCR machine (Biotron healthcare P. Ltd.) using Platinum PCR supermix (11306–016; Life Technologies, USA) following the manufacturer’s instruction. Table 1, depicts the details of the name of genes, the sequence of sense and anti-sense primer, specific annealing temperature and product length.PCR reaction was performed as initial denaturation at 94°C for 2 min followed by 35 cDNA amplification cycles of denaturation at 94°C for 30 sec, annealing at a primer specific annealing temperature for 30 sec and the final extension at 72°C for 30 sec. The analysis of PCR products was performed using agarose gel electrophoresis onto a 1.8% agarose TBE gel and visualized on a UV transilluminator. qPCR was performed with SYBR green master mix qPCR kit (FNZ416L;Thermo Scientific, USA) and BioRad CFX real-time system as per manufacturer’s instructions. No template control (NTC) was placed with each reaction set up for checking any contamination in reaction components, if present. In brief, the master mix had 10 μl of SYBR green, 0.5 μl of each forward and reverse primer, 8 μl NFW and 1 μl of cDNA. The real-time reaction was performed for 40 cycles with following cycling conditions: denaturation at 95°C for 30 sec, annealing at primer specific annealing temperature for 15 sec and extension at 72°C for 30 sec. The data for each gene was normalized using RPS15A as an internal control. The relative quantification of mRNA expression profile for each gene was determined by the equation suggested by Pfaffl, [36]. MSCs derived from cAF at P3 were used as endogenous control for relative expression analysis.

**Table 1:** Gene specific oligonucleotide primers for PCR amplification.

Gene	Primer Pairs (5’-3’);(3’-5’)	Annealing temp (°C)	Amplicon size (bp)	References
CD73	**F-**CTGAGACACCCGGATGAGAT; **R-**ACTGGACCAGGTCAAAGGTG	55	160	[37]
CD90	**F-**GTGAACCAGAGCCTTCGTCT;**R-**GGTGGTGAAGTTGGACAGGT	55	201	[37]
CD105	**F-** ACAAAGGCCTCGTCCTACCT;**R-** TGTGGTTGGTGCTACTGCTC	59	177	[37]
CD34	**F**-CAGCCTCTACGATGTCTC;**R**-GTAATAATGGAAGAAGTCACA	60	276	[38]
Oct-4	**F-**GAGCCGAACCCTGAGGA;**R-**AGGGTAAGCCCCACATCG	60	125	NM_001285569.1
Sox-2	**F-**CTATGACCAGCTCGCAGAC;**R-**ACTTCACCACCGAGCCCA	60	111	[37]
Nanog	**F-**GCAGGTGAAGACCTGGTTC;**R-**CCACATGGGCAGGTTTCCA	60	175	[37]
KLF4	**F-**AGTTCTCATCTCAAGGCACAC;**R-**GTAGTGCCTGGTCAGTTCATC	58	114	XM006079409.1
cMyc	**F-**GAAAAAGCCCCCAAGGTAGTTATC;**R-**TTAGGCGCAAGAGTTCCGTATC	58	159	XM06074649.1
RPS 15A	**F-** AATGGTGCGCATGAATGTC;**R-**GACTTTGGAGCACGGCCTAA	60	100	[39]

### Statistical analysis

Statistical analysis in between different fetal adnexa derived MSCs was carried out by one-way ANOVA with Duncan post hoc test using SPSS software 17.0 (SPSS Inc., Chicago, IL, USA) and differences with (P<0.05) were considered as statistically significant. All the data has been presented as mean ± S.E.M.

## Results

The present study was conducted to compare the isolation, *in vitro* cell expansion along with phenotypic characterization and the *in vitro* tri-lineage differentiation of caprine fetal adnexa derived MSCs.

### Culture characteristics and growth kinetics of caprine fetal adnexa MSCs

**Fig 1:**
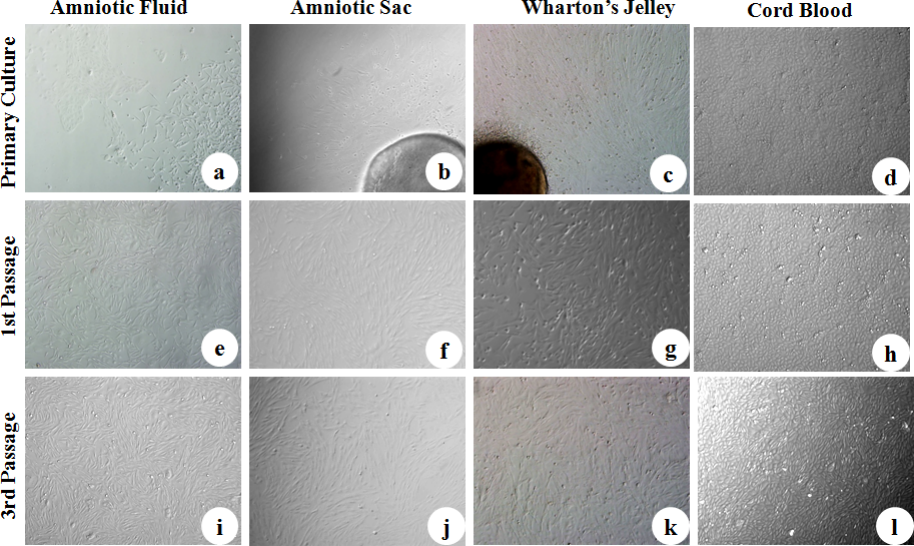
Culture and expansion of caprine fetal adnexa derived MSCs observed under bright field microscope. a) Amniotic Fluid, b) Amniotic Sac, c) Wharton’s Jelly and d) Cord Blood monolayer represents the primary culture. (e),(f),(g) and (h) represents the 1^st^ passage of respective cell lineages. (i),(j),(k) and (l) represents the 3^rd^ passage of respective cell lines. (Magnification-10X).

Culture and expansion of MSCs derived from caprine fetal adnexa observed under bright field microscope have been presented in Fig 1. Surface attachment ability was the major criteria for selection of cells. cWJ explants and cAF mononuclear cells attached to the surface within 48–72 hrs whereas cAS explants and cCB mononuclear cells took relatively longer time of 96–120 and 144–168 hrs, respectively. Morphologically these spindle-shaped cells resembled typical fibroblasts; cWJ and cAS started migrating away from explant within 72–96 hrs and reached 70% confluence in 8–10 and 10–12 days, respectively. In a few replicates of cWJ and cAS culture, polygonal or small round cells were also observed, more prominently in cells derived from cAS. A mixed population of cells i.e. round, pentagonal and fibroblast type were noticed in the primary cultures of cAF, achieving 70–80% confluence approximately in 10–12 days, however, later passages were predominated by the fibroblastic cells. Polygonal cells of cCB took 2 weeks to reach 70–80% confluence, with a much faster growth thereafter.

**Fig 2:**
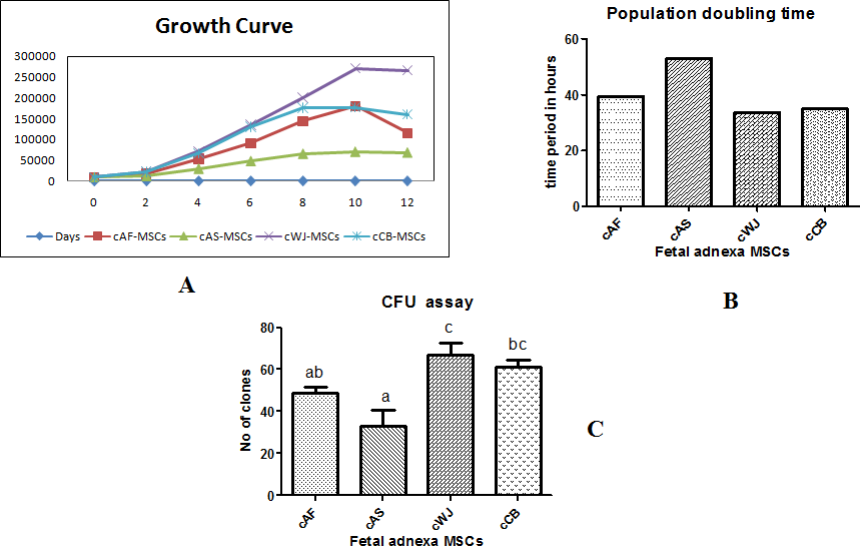
**(A)** Calibrated growth curve plotted for caprine fetal adnexa derived MSCs at P3. **(B)** Population doubling time for caprine fetal adnexa derived MSCs at P3. **(C)** CFU assay for caprine fetal adnexa derived MSCs at P3. Data represents the mean±SEM. Means bearing different superscripts differ significantly (P<0.05) in different cell lines.

The calibrated growth curve (Fig 2A) was plotted for fetal adnexa MSCs at P3. All the four cell lines exhibited an initial lag phase of 36–48 hrs, thereafter exponential growth of 4–6 days occurred for cAF, cWJ and cCB MSCs followed by plateau phase with declined growth rate. The growth rate of cAS MSCs was lower with a short exponential growth than the other three cell lines. The PDT of cAF, cAS, cWJ and cCB MSCs for P3 cells was 39.45, 53.11, 33.70 and 34.98 hrs, respectively (Fig 2B). cWJ and cCB MSCs grew rapidly and had lowest PDT whereas it was highest for cAS MSCs.

The number of cell colonies in each cell line was counted at P1 after seeding 100 cells per petri dish (94X16 mm). Fig 2C highlights the average clones for cAF, cAS, cWJ and cCB were 49, 33, 67 and 61, respectively. The CFU was significantly highest for cWJ and lowest for cAS MSCs (P<0.05).

### Alkaline Phosphatase activity

**Fig 3:**
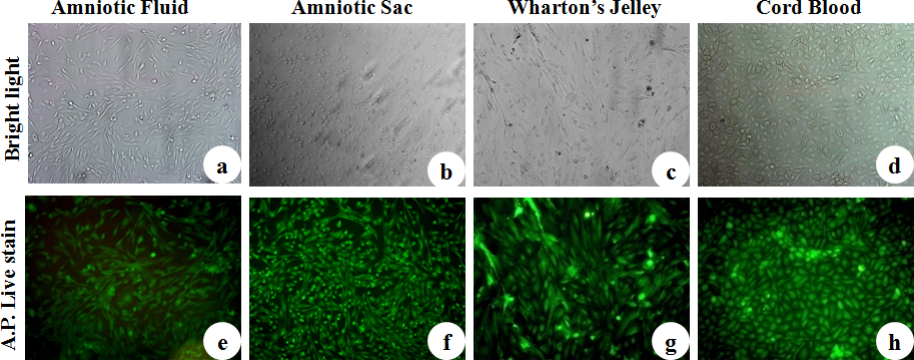
Alkaline Phosphatase staining of caprine fetal adnexa MSCs. a) Amniotic Fluid, b) Amniotic Sac, c) Wharton’s Jelly and d) Cord Blood monolayer under bright field. e) Amniotic Fluid, f) Amniotic Sac, g) Wharton’s jelly and h) Cord Blood monolayer positive for AP stain (Magnification-20X).

The AP activity was assessed for all four cell lines of caprine fetal adnexa MSCs at P3 and was confirmed through AP stain as depicted in Fig 3.

### Chondrogenic differentiation

**Fig 4:**
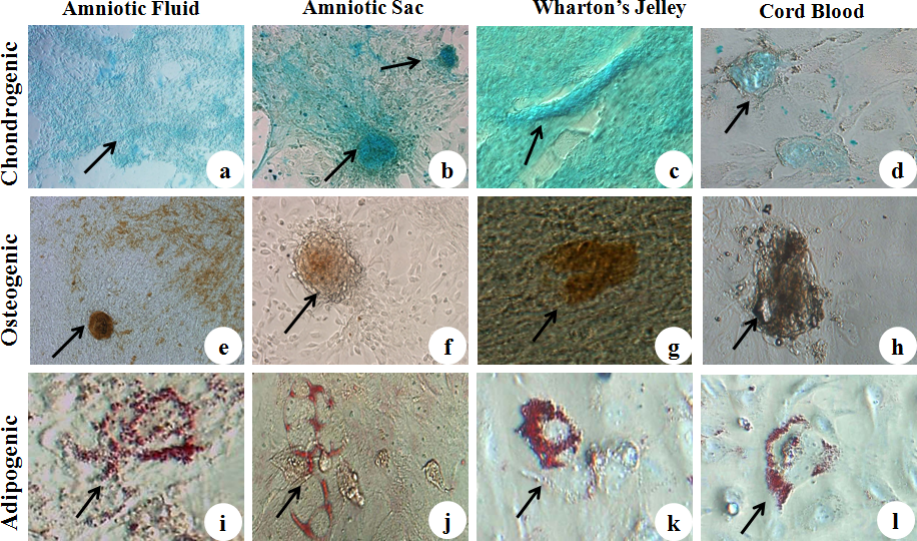
Multi-lineage differentiation of caprine fetal adnexa MSCs. a) Amniotic Fluid, b) Amniotic Sac, c) Wharton’s Jelly and d) Cord Blood monolayer subjected to chondrogenic differentiation. e) Amniotic Fluid, f) Amniotic Sac, g) Wharton’s Jelly and h) Cord Blood monolayer subjected to osteogenic differentiation. i) Amniotic Fluid, j) Amniotic Sac, k) Wharton’s Jelly and l) Cord Blood monolayer subjected to adipogenic differentiation (Magnification-40X).

Chondrogenic differentiation of caprine fetal adnexa derived MSCs was confirmed after 14 days of induction and secretion of cartilage-specific proteoglycans stainable with Alcian blue staining. Fig 4A, 4B, 4C and 4D), depicts the staining characteristics, confirming that cWJ MSCs formed cell aggregateswhich stained more intensely with Alcian Blue whereas cCB MSCs occasionally changed to peripheral cartilaginous differentiationwith mild staining as compared to other cell lines.

### Osteogenic differentiation

Induction of caprine fetal adnexa MSCs to osteogenic differentiation medium resulted in morphological changes observed at 96 hrs. All four cells lines formed distinct cell clusters and secreted extracellular calcium crystals after 14 days of incubation which was demonstrated through dark brown color on Von Kossa staining [Fig 4E, 4F, 4G and 4H)]. cAF MSCs displayed relatively higher osteogenic potential as these cells differentiated earlier and stained more intensely in a dispersed manner than the other three cell lines.

### Adipogenic differentiation

On induction of caprine fetal adnexa MSCs to the adipogenic differentiation medium, MSCs of all four cell lines appeared swollen, roundand these adipocytes burst open after 72–96 hrs. The bright red lipid-laden adipocytes were evidenced by oil red o staining after 14 days of induction as shown in Fig 4I, 4J, 4K and 4l).

### Phenotypic characterization

A panel of positive surface markers for MSCs along with a negative marker and a battery of pluripotency markers were localized by immunofluorescence, flow cytometric analysis and further confirmed by real-time PCR.

**Fig 5:**
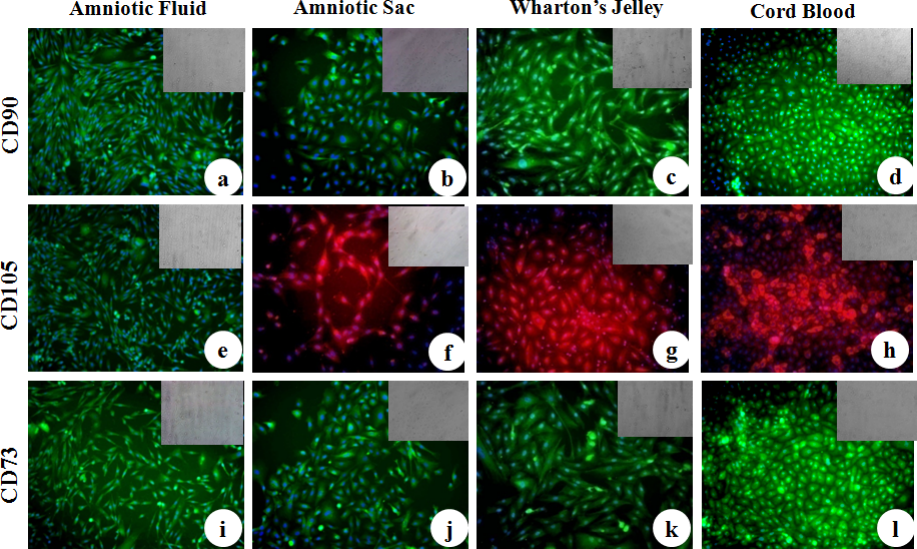
Immunocytochemical localization of cell surface antigens in caprine fetal adnexa MSCs. a) Amniotic Fluid, b) Amniotic Sac, c) Wharton’s Jelly and d) Cord Blood monolayers stained with primary antibodies directed against CD90. e) Amniotic Fluid, f) Amniotic Sac, g) Wharton’s Jelly and h) Cord Blood monolayer stained with primary antibodies directed against CD105. i) Amniotic Fluid, j) Amniotic Sac, k) Wharton’s Jelly and l) Cord Blood monolayer stained with primary antibodies directed against CD73 (Magnification-20X).

**Fig 6:**
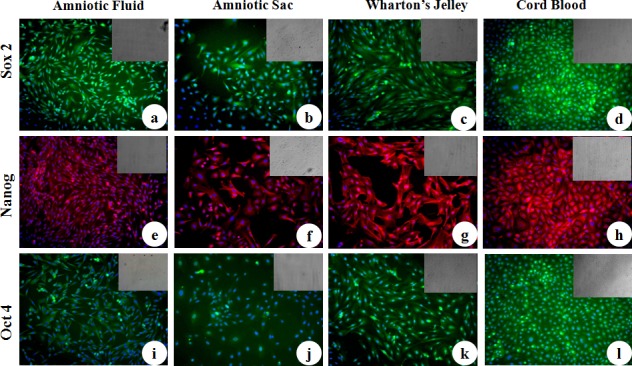
Immunocytochemical localization of pluripotency markers in caprine fetal adnexa MSCs. a) Amniotic Fluid, b) Amniotic Sac, c) Wharton’s jelly and d) Cord Blood monolayers stained with primary antibodies directed against Sox2. e) Amniotic Fluid, f) Amniotic Sac, g) Wharton’s Jelly and h) Cord Blood monolayer stained with primary antibodies directed against Nanog. i) Amniotic Fluid, j) Amniotic Sac, k) Wharton’s Jelly and l) Cord Blood monolayer stained with primary antibodies directed against Oct4 (Magnification-20X).

**Fig 7:**
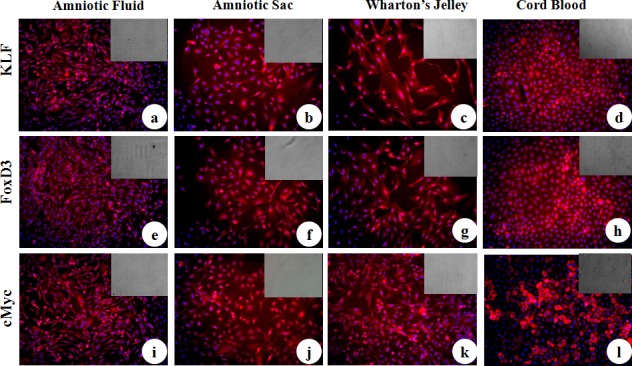
Immunocytochemical localization of pluripotency markers in caprine fetal adnexa MSCs. a) Amniotic Fluid, b) Amniotic Sac, c) Wharton’s Jelly and d) Cord Blood monolayers stained with primary antibodies directed against KLF. e) Amniotic Fluid, f) Amniotic Sac, g) Wharton’s Jelly and h) Cord Blood monolayer stained with primary antibodies directed against FoxD3. i) Amniotic Fluid, j) Amniotic Sac, k) Wharton’s Jelly and l) Cord Blood monolayer stained with primary antibodies directed against cMyc (Magnification-20X).

**Fig 8:**
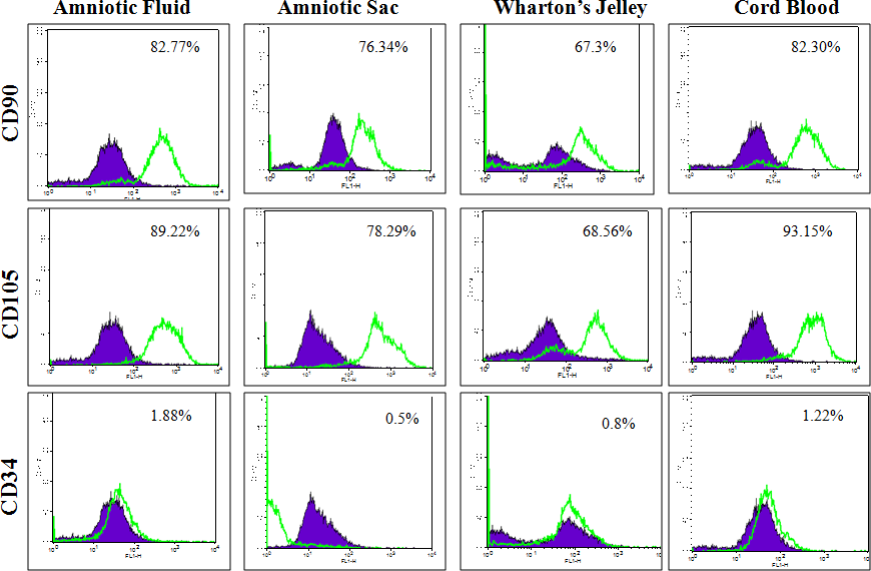
Overlay histogram of flow cytometric analysis of cell surface markers CD73, CD105, CD34. The empty histogram represents the analysis with mAbs in MSCs.

**Fig 9:**
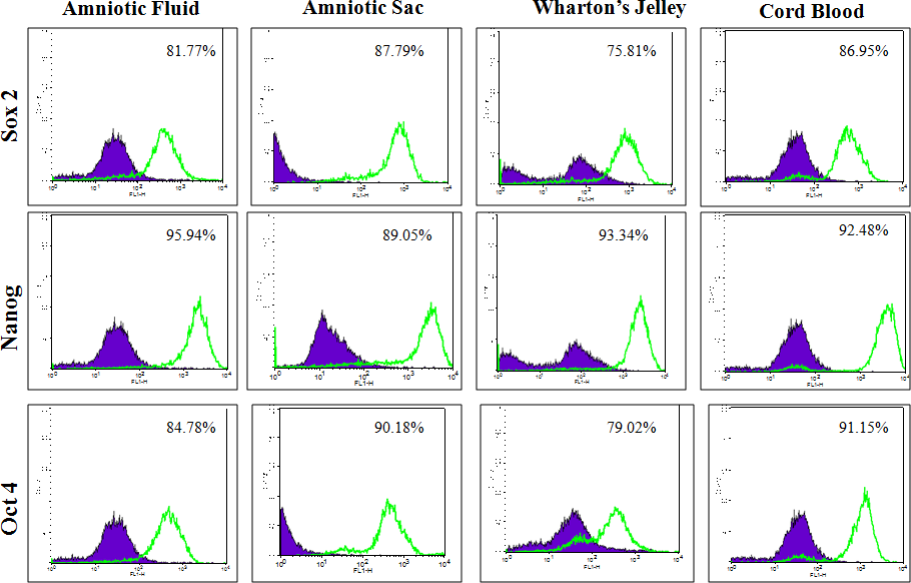
Overlay histogram of flow cytometric analysis of pluripotency markers Oct4, Sox2 and Nanog. The empty histogram represents the analysis with mAbs in MSCs.

MSC cell surface antigens (CD73, CD90 and CD105) were localized in cWJ, cAS, cAF and cCB monolayers by positive immunofluorescence staining (Fig 5). Pluripotency markers (Oct4, Sox2, Nanog, KLF, cMyc and FoxD3) were also localized in all the fetal adnexa MSC monolayers (Figs 6 and 7). Flow cytometric analysis could not discern any distinct characteristics exhibited by MSC population derived from different tissues. All the four cell lines were negative for the HSC marker CD34 (less than 2% in each cell line) and positive for CD90 and CD105 (Fig 8). All the four fetal adnexa MSCs were also positive for pluripotency markers i.e. Oct4, Sox2 and Nanog (Fig 9).

#### Expression profile of surface and pluripotency markers

**Fig 10:**
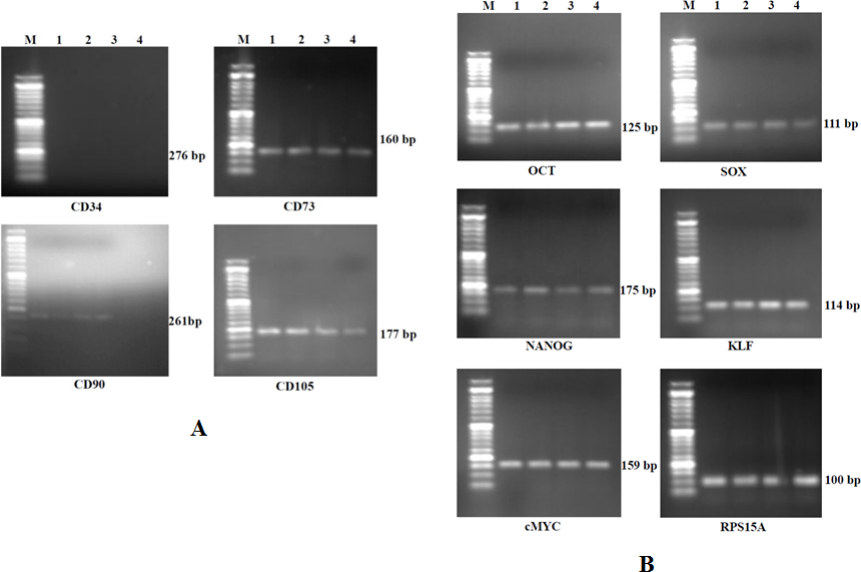
**(A)** Gel electrophoresis picture of cell surface antigens (CD73, CD90 and CD105) and negative marker (CD34). Lane M: 50 bp Ladder; Lane 1: cAF-MSCs; Lane 2: cAS-MSCs; Lane 3: cWJ-MSCs; Lane 4: cCB-MSCs. **(B)** Gel electrophoresis picture of pluripotency markers (Oct4, Sox2, Nanog, KLF, cMyc) and endogenous control (RPS15A). Lane M: 50 bp Ladder; Lane 1: cAF MSCs; Lane 2: cAS MSCs; Lane 3: cWJ MSCs; Lane 4: cCB MSCs.

RelativemRNA expression of all the four fetal adnexa MSCs was done at P3. All the four lines expressed surface antigens i.e.CD73, CD90 and CD105 [Fig 10A] whereas, they did not express CD34 (negative marker). All the four fetal adnexa MSCs also expressed the pluripotency markers i.e.Oct4, Sox2, Nanog, KLF and cMyc [Fig 10B].

**Fig 11:**
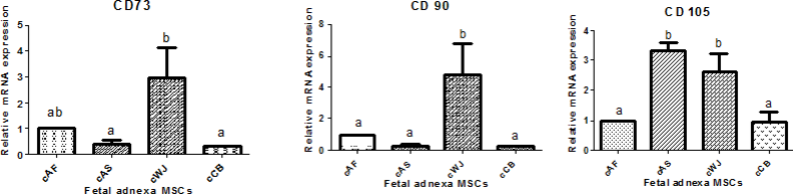
Relative mRNA expression of cell surface antigens (CD73, CD90 and CD105) in caprine fetal adnexa MSCs at P3. Data represents the mean±SEM. Means bearing different superscripts differ significantly (P<0.05) in different cell lines.

**Fig 12:**
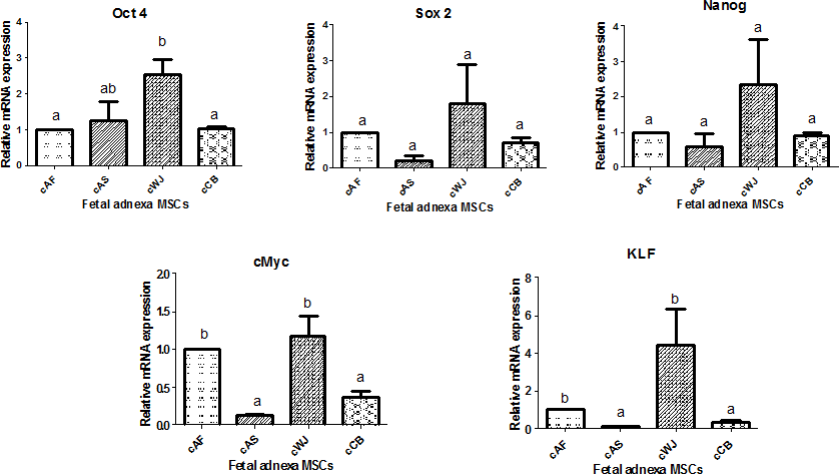
Relative mRNA expression of pluripotency markers(Oct4, Sox2, Nanog, KLF, cMyc) in caprine fetal adnexa MSCs at P3. Data represents the mean±SEM. Means bearing different superscripts differ significantly (P<0.05) in different cell lines.

The relative mRNA expression of the surface antigens (CD73, CD90 and CD105) in caprine fetal adnexa derived MSCs at P3 are presented in Fig 11.The mRNA expression of CD73 was found significantly (P<0.05) higher in cWJ MSCs as compared to cAS and cCB MSCs but no significant difference (P<0.05) was found with cAF MSCs. On the other hand, mRNA expression of CD90 was significantly (P<0.05) higher in cWJ MSCs as compared to the other fetal adnexa MSCs but no significant difference was observed between cAF, cAS and cCB MSCs. The relative mRNA expression of CD105 was significantly (P<0.05) higher in cWJ and cAS MSCs than in cAF and cCB MSCs but there was no significant difference between cWJ and cAS MSCs. The relative mRNA expression of pluripotency markers (Oct4, Sox2, Nanog, KLF and cMyc) in caprine fetal adnexa derived MSCs at P3 are presented in Fig 12.The mRNA expression of Oct4 was found significantly (P<0.05) higher in cWJ MSCs as compared to cAF and cCB MSCs but no significant difference (P<0.05) was found with cAS MSCs. The mRNA expression of Sox2 and Nanog did not differ significantly among different caprine fetal adnexa derived MSCs. The relative mRNA expression of KLF and cMyc was found to be significantly (P<0.05) higher in cWJ and cAF MSCs than in cAS and cCB MSCs but did not differ significantly between cWJ and cAF MSCs.

## Discussion

In the present study, caprine MSCs derived from different compartments of fetal adnexa were characterized with respect to *in vitro* growth kinetics, expression of characteristic molecular markers and differentiation potential. Naive fetal adnexa MSCs were obtained from 2–3 months old caprine fetuses, as in many mammalian species, much of the placental growth occurs during the first half of gestation [40].

### Morphological Characteristics

The *in vitro* expansion of all the fetal adnexa cell lines depicted variable cellular morphologies. The primary culture of cAF was observed to have mixed population of cells whereas subsequent passages had predominantly fibroblastic cells. These observations corroborate the findings in caprines [29], bovines [34, 41–42] and humans [43–44]. Our findings are at variance with reports on bovine and porcine species. According to some reports, in the bovine [45] and porcine [46] species, round and fibroblastic cells persisted throughout passages. This heterogeneous population of cells is believed to originate from fetal skin, urine, fetal membranes and mesenchymal tissues [43–44].

Caprine WJ-derived stem cells depicted a typical fibroblast type of colonies with occasional polygonal or small round cells. This agrees with previous reports on caprine [28, 30], bubaline [37] and equine [32] species, however, some researchers reported heterogeneous cell population in porcine [47] and caprine [27] species.

The MSCs derived from cAS also depicted fibroblast morphology along with polygonal or small round cells. In contrast, bovine AS MSCs displayed only polygonal morphology [34]. In this study, cCB MSCs depicted polygonal morphology at all the passages, however, in equines [32, 48] and canines [49], spindle-like fibroblast cells were observed and in felines, a heterogeneous population were observed [50]. This suggests that morphological characters of fetal adnexa derived MSCs are typical for a given species and this variation may be due to the difference in gestational length or the period of gestation at which the tissues are derived. Other reasons for variation could be due to differences in culture media constituents, FBS concentration, adherence to plastic surfaces and tissue harvesting techniques [51].

### Growth Kinetics

The growth curve of all four cell lines depicted three distinct phases; initial lag phase followed by exponential growth and finally the plateau phase, which is in agreement with the general behavior of the MSCs [52]. In the present study, the exponential growth was comparable for cAF, cWJ and cCB but the growth rate of cAS MSCs was slower with a shorter exponential growth phase. It was also observed that cWJ MSCs expanded more rapidly with faster PDT and higher clonogenic potential than other fetal adnexa MSCs. This is supported by similar observations in equines [32] and humans [53, 54] but in canine species, AF MSCs showed faster growth rate than the other fetal adnexa MSCs [31].

### Tri-lineage differentiation

In the present study, tri-lineage differentiation potential was demonstrated based on the principles of International Society for Cellular Therapy (ISCT) [55]. All the four cell lines differentiated into chondrogenic, osteogenic and adipogenic lineages, but cWJ MSCs displayed higher chondrogenic and adipogenic potential making it a suitable candidate for cartilage injuries. On the other hand cAF MSCs depicted higher osteogenic potential than other MSCs, hence, may be a better option for use in fracture and bone related injuries. Contrary to the above findings, equine CB MSCs displayed higher adipogenic potential [32]. Although the tri-lineage differentiation ability of fetal adnexa derived MSCs have been demonstrated previously in caprine [29, 30], equine [32, 56–59], canine [31, 49], bovine [34, 42, 60–61] and bubaline [37] species but except for equine species, a comparative evaluation of multi-lineage differentiation is not available for other species of animals.

### MSC surface markers

All the four caprine fetal adnexa MSCs were CD73^+^, CD105^+^, CD90^+^ and CD34^-^. The expression of surface markers have been reported in cWJ and cAF MSCs [30] however; there are no reports on these markers in caprine AS and CB MSCs. The expression of surface antigen markers in AF [42], WJ [37, 59, 62], AS with debatable CD73 expression [56, 58] and CB [63, 64] MSCs have been reported in various species, though not in a comparative mode. There are several reports on positive markers; with each research group using a different subset of markers but our criteria of selection was based on principles of ISCT [55]. The relative mRNA expression of these markers depicted significant variation at P3. The mRNA expression of CD73 and CD90 was significantly higher in cWJ cells as compared to the other fetal adnexa derived MSCs suggesting its better ability for cell adhesion, cell-cell and cell-matrix interactions along with greater nerve regeneration ability and neurite outgrowth [65, 66]. The relative mRNA expression of CD105 was observed to be significantly higher in cWJ and cAS MSCs, hence, may have a higher angiogenic potential [67].

### Pluripotency markers

The caprine fetal adnexa MSCs were positive for the pluripotency markers (Oct4, Sox2, Nanog, KLF and cMyc) at P3. According to previous reports, WJ MSCs derived from caprine, bubaline, porcine and equine species were positive for Oct4, Sox2 and Nanog [18,30, 37, 47, 68], however in canines, P2 onwards the Oct4 expression was lost and expression of Nanog and Sox2 decreased [31, 62]. AF MSCs are also reported to be Oct4^+^, Sox2^+^ and Nanog^+^ in caprine, ovine and canine species [29, 69–70]however, a study in bovine AF MSCs reported the absence of Oct4, Sox2 and Nanog [45]. Nanog expression in AS derived MSCs varies considerably between species. It is expressed in equine [71], bubaline [72] but not in canine AS MSCs [31, 73], however, all the above-mentioned species are Oct4^+^ and Sox2 ^+^. CB MSCs are reported to be Oct4^+^[49, 60, 64, 74], Nanog^+^ and Sox2^+^ [64, 74]. But some studies reported an absence of pluripotency markers in CB MSCs [48, 75]. KLF has been localized in human AS MSCs [76]. All these pluripotency markers are transcriptional factors maintaining the cells in a self-renewal state. The relative mRNA expression of KLF and cMyc was observed to be significantly higher in cWJ and cAF MSCs than the other two cell lines at P3, but no significant differences were observed in the expression of Sox2 and Nanog. The mRNA expression of Oct4 was significantly higher in cWJ than cAF and cCB MSCs but no significant difference was found when compared with cAS MSCs. These results further confirmed that WJ MSCs have a better self-renewal capacity and hence grow faster with a longer exponential phase than the other cells lines. Further studies are required for a better understanding of the variability in mRNA expression of surface and pluripotency markers in the four cell lines derived from fetal adnexa.

The present study provides an insight into the relative performance of caprine MSCs derived from different sources of fetal adnexa and specifies the best performer in terms of growth kinetics, tri-lineage differentiation and expression of surface and pluripotency markers. However, in-depth studies are required on immunomodulatory properties and preclinical trials to explore the scope of caprine fetal adnexa derived MSCs in veterinary regenerative medicine and *in vivo* application.

## Conclusion

In summary, all the four cell lines of caprine fetal adnexa MSCs stained positively to AP, differentiated into osteogenic, chondrogenic and adipogenic lineages; expressed characteristic MSC surface antigens and pluripotency markers. The comparative assessment indicated that cWJ MSCs had better growth characteristics and higher mRNA expression of MSC surface antigens and pluripotency markers in comparison to cAS, cAF and cCB. These findings also suggest that caprine MSCs derived from fetal adnexa represents readily obtainable and highly proliferative non-invasive stem cell source for regenerative therapeutics.
